# The Impact of Low-Dose Insulin on Peripheral Nerve Insulin Receptor Signaling in Streptozotocin-Induced Diabetic Rats

**DOI:** 10.1371/journal.pone.0074247

**Published:** 2013-08-30

**Authors:** Kazuhiro Sugimoto, Masayuki Baba, Susumu Suzuki, Soroku Yagihashi

**Affiliations:** 1 Department of Laboratory Medicine, Hirosaki University Graduate School of Medicine, Hirosaki, Japan; 2 Diabetes Center, Ohta Nishinouchi Hospital, Koriyama, Japan; 3 Department of Neurology, Aomori Prefectural Central Hospital, Aomori, Japan; 4 Department of Pathology and Molecular Medicine, Hirosaki University Graduate School of Medicine, Hirosaki, Japan; Consiglio Nazionale delle Ricerche, Italy

## Abstract

**Background:**

The precise mechanisms of the neuroprotective effects of insulin in streptozotocin (STZ)-induced diabetic animals remain unknown, but altered peripheral nerve insulin receptor signaling due to insulin deficiency might be one cause.

**Methodology and Principal Findings:**

Diabetes was induced in 10-week-old, male Wistar rats by injecting them with STZ (45 mg/kg). They were assigned to one group that received half of an insulin implant (∼1 U/day; I-group, n = 11) or another that remained untreated (U-group, n = 10) for 6 weeks. The controls were age- and sex-matched, non-diabetic Wistar rats (C-group, n = 12). Low-dose insulin did not change haemoglobin A1c, which increased by 136% in the U-group compared with the C-group. Thermal hypoalgesia and mechanical hyperalgesia developed in the U-group, but not in the I-group. Sensory and motor nerve conduction velocities decreased in the U-group, whereas sensory nerve conduction velocity increased by 7% (*p* = 0.0351) in the I-group compared with the U-group. Western blots showed unaltered total insulin receptor (IR), but a 31% decrease and 3.1- and 4.0-fold increases in phosphorylated IR, p44, and p42 MAPK protein levels, respectively, in sciatic nerves from the U-group compared with the C-group. Phosphorylated p44/42 MAPK protein decreased to control levels in the I-group (*p*<0.0001).

**Conclusions and Significance:**

Low-dose insulin deactivated p44/42 MAPK and ameliorated peripheral sensory nerve dysfunction in rats with STZ-induced diabetes. These findings support the notion that insulin deficiency per se introduces impaired insulin receptor signaling in type 1 diabetic neuropathy.

## Introduction

Insulin acts as a potent neurotrophic factor that specifically stimulates neurite outgrowth and regeneration of sensory neurons [1], [2]. There is evidence that insulin deficiency rather than hyperglycemia may be involved in the pathogenesis of type 1 diabetic neuropathy [3], [4]. In streptozotocin (STZ)-induced diabetic rats, which is the most extensively studied animal model of type 1 diabetic neuropathy, low-dose insulin insufficient to affect systemic glycemia reverses abnormal peripheral nerve function and structure [5]–[9]. A more recent study also showed that intraplantar injections of low-dose insulin resulted in rapid improvement of epidermal innervation in STZ-induced diabetic mice without altering innervation of the opposite paw [10]. We have demonstrated that the high-affinity insulin receptor is expressed preferentially in small-to-medium-sized sensory neurons in rats [11], [12] and that impaired peripheral nerve insulin receptor signaling, as indicated by decreased expression of phosphorylated insulin receptor, occurs during the early course of altered pain sensation in STZ-induced diabetic rats [13]. However, the effects of low-dose insulin on impaired peripheral nerve insulin receptor signaling in association with altered nociception in type 1 diabetic neuropathy remain to be explored. This information would be particularly important to better understand the potential role of impaired peripheral nerve insulin signaling in the pathogenesis of diabetic neuropathy and provide new ideas on their underlying mechanisms that the glycaemic hypothesis cannot otherwise fully explain [14], [15]. Therefore, in the present study, the impacts of chronic administration of low-dose insulin on neuronal function and structure, as well as on the neuronal expression and localization of insulin receptor signaling molecules including phosphorylated insulin receptor, Akt, and mitogen-activated protein kinase (MAPK), were investigated in STZ-induced diabetic rats.

## Methods

### Experimental animals

All animal protocols were approved by the Animal Research Committee and followed the Guidelines for Animal Experimentation of Hirosaki University (M08088) as well as the Principles of laboratory animal care (NIH publication no. 85–23, revised 1985). All surgery was performed under sodium pentobarbital anesthesia, and all efforts were made to minimize suffering. Diabetes was induced in 10-week-old, male Wistar rats (n = 21; Clea Japan, Tokyo, Japan) by injecting them with STZ (45 mg/kg). Six weeks after the induction of diabetes**,** they were assigned to one group that received half of an insulin implant (∼1 U/day) (Linplant; LinShin Canada, Inc., Scarborough, Canada) (n = 11) or another that remained untreated (n = 10) for 6 weeks. The implant was placed in the subcutaneous tissue located on the back following the manufacturer's instructions (http://www.linshincanada.com/linbit.html) under anaesthesia with pentobarbital (40 mg/kg). The controls were age- and sex-matched, non-diabetic, Wistar rats (n  = 12). All animals were housed in sawdust in plastic cages. The rats were maintained on a 12-h/12-h, light/dark cycle at 22°C±2°C and 55%±5% relative humidity and allowed free access to tap water and standard laboratory chow (MF; Oriental Yeast Co., Ltd., Tokyo, Japan). The animals were weighed, and blood glucose levels were monitored regularly. Whole blood glucose levels of tail vein samples obtained between 14:00 and 16:00 were measured using an Accu-Chek Compact Plus blood glucose meter (Roche Diagnostics K.K., Tokyo, Japan). At the end of the experiment, non-fasting animals were killed by exsanguination from the left cardiac ventricle under deep anaesthesia with pentobarbital (∼100 mg/kg) between 10:00 and 16:00. Then, blood samples were collected to determine hemoglobin A1c, serum lipid, insulin, adiponectin, and leptin levels, as described previously [16].

### Behavioural tests of nociception

Before starting the experiments, the animals were allowed to acclimatize to handling-related stress for at least 1 week. At the indicated time points, tail-flick latency (TFL) and the mechanical nociceptive threshold (MNT) of each animal were determined to assess nociceptive responses to noxious thermal and pressure stimuli, respectively. The MNT was assessed with the Randall-Selitto test using an Analgesymeter (Ugo-Basile, Varese, Italy). A constantly increasing pressure stimulus (with increase at a rate of 16 g/s) was applied to the dorsal surface of the rat hind paw while the animal was gently restrained under a soft towel; to avoid tissue damage, a cutoff of 250 g was used. The pressure was increased until the animal withdrew the paw, squeaked, or struggled. One measurement per paw was performed with an interval of longer than 15 minutes between measurements; for each animal, the results for the 2 paws were averaged for use in statistical analysis. Thermal sensitivity was assessed using a Tail Flick Analgesymeter (MK-330B; Muromachi Kikai Co, Ltd., Tokyo, Japan). The animal was gently wrapped in a towel and placed on the top of the instrument with the tail in the sensing groove. The tail flick latency was determined by exposing the animal’s tail to a radiant heat source and recording the time taken to remove the tail from the noxious thermal stimulus. The radiation intensity was chosen on the basis of the intensity required to elicit a basal tail flick response of 4 to 8 seconds in control rats. For each animal, 2 to 3 recordings were made at an interval of longer than 15 minutes; the mean value was used for statistical analysis.

### Nerve conduction studies

At the end of the experiment, nerve conduction was assessed in sciatic-tibial nerves as described [16].

### Morphometric analyses of myelinated fibers

Sural nerves at the level of the lower calf were excised and subjected to morphometric analyses as described previously [17] using Win ROOF image software (Mitani Co., Ltd., Fukui, Japan).

### Intraepidermal nerve fiber density (IENFD)

Hind footpad skin specimens were obtained with a 3-mm punch tool and processed as described previously [16]. Images were collected using a Zeiss LSM510 confocal microscope (Carl Zeiss, Oberkochen, Germany) and a 40× water immersion objective lens. Sixteen confocal images were captured at 2-µm intervals, and the image stack was superimposed to produce an image for quantitation. Images were analysed using Zeiss LSM 510 image browser software (Carl Zeiss) by tracing protein gene product (PGP) 9.5-imunostained intraepidermal nerve fibres in three dimensions. Individual intraepidermal nerve fibres were counted as they passed through the basement membrane. Epidermal nerve counts of PGP 9.5-immunoreactive fibres within the projected 30-µm-thick image stacks are expressed as numbers of fibres/mm of epidermis.

### Lumbar dorsal root ganglion (DRG) immunofluorescence

Bilateral lumbar DRGs (L4) were excised at the end of the experiment and processed as described previously [16]. Cryostat sections (14-µm-thick) of DRGs were double immunostained using a mouse monoclonal primary antibody against actin (1∶200; Sigma-Aldrich, St. Louis, MO, USA) and a rabbit polyclonal primary antibody against phosphorylated insulin receptor (1∶200; Abcam, Cambridge, UK) or a rabbit monoclonal primary antibody against phosphorylated p44/42 MAPK (Thr202/Tyr204) (1:200; Cell Signaling Technology, Beverly, MA, USA). These antigens were localized with Alexa 568-conjugated anti-mouse IgG (1∶200, Molecular Probes) and Alexa 488-conjugated anti-rabbit IgG (1∶200, Molecular Probes). The sections were cover-slipped with Prolong Gold antifade reagent containing DAPI (Molecular Probes) to avoid photo-bleaching and were examined by confocal microscopy (Carl Zeiss) and a 20× objective lens at identical settings. The negative controls were sections stained without primary antibodies. Actin immunoreactivity served as a control to ensure that the background signals were comparable among all groups. In addition, the scanning conditions including contrast, brightness level and pinhole size were set constant throughout the whole observation period for both control and diabetic samples. Over 100 neurons with clearly identifiable nuclei per animal were analysed with respect to neuronal area and mean pixel intensity values of the selected neurons using Image J software (National Institutes of Health, Bethesda, MD, USA), and the results for each animal were averaged for statistical analysis. Intensity was plotted against neuronal area to compare between the groups. Observers were blinded to the experimental groups.

### Western blotting

The left sciatic nerve was collected from each animal and subjected to Western blot analysis as described previously [16] using the primary antibodies listed below: mouse monoclonal primary antibodies against IR (beta-subunit) (Santa Cruz Biotechnology, Santa Cruz, CA, USA), p44/42 MAPK (Cell Signaling), and actin (Sigma-Aldrich); rabbit polyclonal primary antibodies against phosphorylated insulin receptor (pY972) (Biosource, Camarillo, CA, USA), IRS-1, phosphorylated IRS-1 (Ser302), IRS-2, Akt, and phosphorylated Akt (Ser473) (Cell Signaling); rabbit monoclonal primary antibodies against phosphorylated p44/42 MAPK (Thr202/Tyr204) (Cell Signaling).

### Data analysis

All data were statistically analysed using Stat View software (version 4.5). Values are described as means ± SE. The significance of differences in mean values between two groups was tested by ANOVA followed by the Bonferroni/Dunn test. A two-sided *p* value of <0.05 was considered significant.

## Results

### Body weight and laboratory data

At the end of the experiment, the body weight of untreated STZ-induced diabetic rats was less than that of control rats and comparable to that of insulin-treated STZ-induced diabetic rats (Table 1). Hemoglobin A1c, serum total cholesterol, and free fatty acid levels were increased by 136%, 17%, and 40%, respectively, in untreated STZ-induced diabetic rats compared with control rats (Table 1). Serum insulin, adiponectin, and leptin levels decreased by 92%, 43%, and 92%, respectively, in untreated STZ-induced diabetic rats compared with control rats (Table 1). Insulin-treated STZ-induced diabetic rats exhibited equivalent glycemia, adiponectin, leptin, and total cholesterol levels, and a significant 17% decrease in serum free fatty acid levels compared with untreated STZ-induced diabetic rats. Insulin treatment approximately doubled non-fasting serum insulin levels from 0.17±0.03 ng/ml in the untreated diabetic group to 0.34±0.05 ng/ml in the treated group, but the difference between the two groups did not reach statistical significance (Table 1).

**Table 1:** Body weight and laboratory data from control, untreated and insulin-treated STZ-induced diabetic rats at the end of the study.

	Control	Untreated STZ-induced diabetic rats	Insulin-treated STZ-induced diabetic rats
N	12	10	10
Body weight (g)	467±10	339±13^§^	349±4^§^
A1c (%)	3.3±0.1	7.8±0.1^§^	8.1±0.2^§^
Insulin (ng/ml)	2.03±0.51	0.17±0.03^‡^	0.34±0.05^†^
Adiponectin (µg/ml)	4.4±0.4	2.5±0.2^‡^	3.1±0.2^*^
Leptin (ng/ml)	7.2±1.2	0.6±0.1^§^	1.1±0.2^§^
T-chol (mg/dl)	84.3±3.4	98.5±5.0^*^	94.3±3.7
Free fatty acid (µEq/l)	701±38	978±54^†^	807±74^**^

Data are means ± SE. ^*^ *p*<0.05, ^†^ *p*<0.005, ^‡^ *p*<0.0005, and ^§^ *p*<0.0001 vs. control rats; and ^**^ *p*<0.05 vs. untreated STZ rats. T-chol: total cholesterol.

### Behavioural tests of nociception

**Figure 1:**
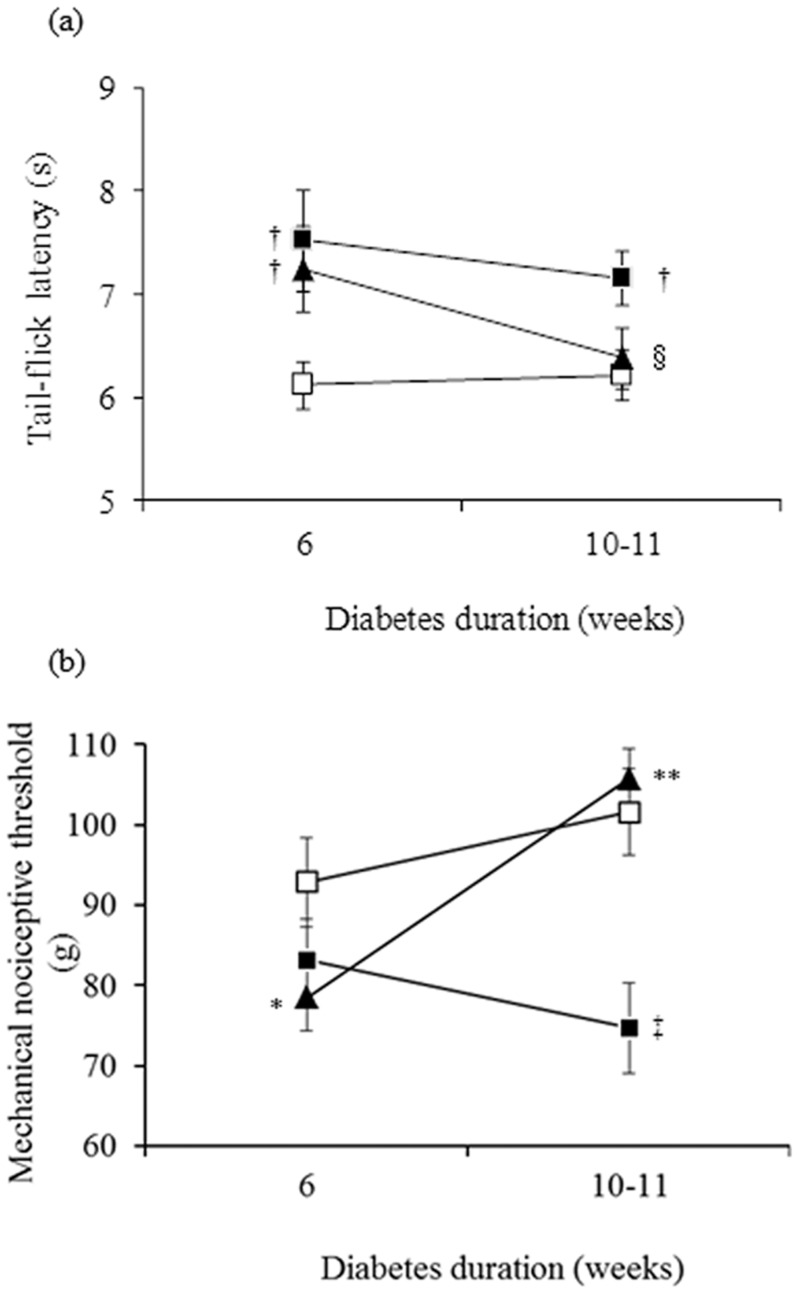
A low dose of insulin for 4 to 5 weeks normalizes impaired thermal (a) and mechanical (b) perception in diabetic rats. Nociceptive responses to thermal and mechanical stimuli are expressed as tail-flick latency (seconds) and paw-pressure withdrawal threshold (grams), respectively, in control (white squares), untreated STZ-induced diabetic (black squares) and insulin-treated STZ-induced diabetic (black triangles) rats. Data are means ± SE of 11 control, 9 to 10 untreated STZ-induced diabetic and 11 insulin-treated STZ-induced diabetic rats for tail-flick latency, and of 8 to 10 control, 10 untreated STZ-induced diabetic, and 10 to 11 insulin-treated STZ-induced diabetic rats for paw-pressure withdrawal threshold measurements. ^*^ *p* = 0.05, ^†^ *p*<0.05, and ^‡^ *p*<0.001 vs. control rats; ^§^ *p* = 0.05 and ^**^ *p*<0.0005 vs. untreated STZ-induced diabetic rats.

Six weeks after diabetes was induced in untreated and insulin-treated STZ-induced diabetic rats, TFL to thermal stimuli (Fig. 1a) was increased (thermal hypoalgesia) by 23% (*p*<0.05) and 18% (*p*<0.05), respectively, whereas MNT to pressure stimuli (Fig. 1b) was decreased (mechanical hyperalgesia) by 10% (not significant; *p* > 0.05) and 16% (marginally significant; *p* = 0.05), respectively, compared with control rats. Low-dose insulin administration for 4 to 5 weeks reversed the thermal and mechanical pain sensations.

### Nerve conduction studies

**Figure 2:**
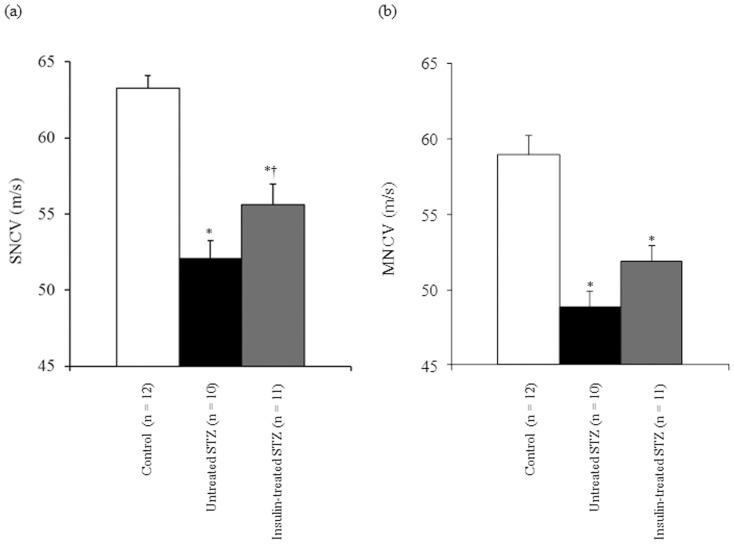
Sensory (SNCV) (a) and motor nerve conduction velocity (MNCV) (b) in left sciatic-tibial nerves of control (white bars), untreated STZ-induced diabetic (black bars), and insulin-treated STZ-induced diabetic (grey bars) rats. Data are means ± SE. ^*^ *p*<0.0001 vs. control rats; ^†^ *p*<0.05 vs. untreated STZ-induced diabetic rats.

In untreated STZ-induced diabetic rats, both SNCV (Fig. 2a) and MNCV (Fig. 2b) were significantly decreased by 18% and 17%, respectively, compared with control rats. In insulin-treated STZ-induced diabetic rats, SNCV increased significantly (*p*<0.05) by 7% and MNCV increased non-significantly (*p* = 0.07) by 6% compared with untreated STZ-induced diabetic rats.

### Morphometric analyses of myelinated fibers

**Figure 3:**
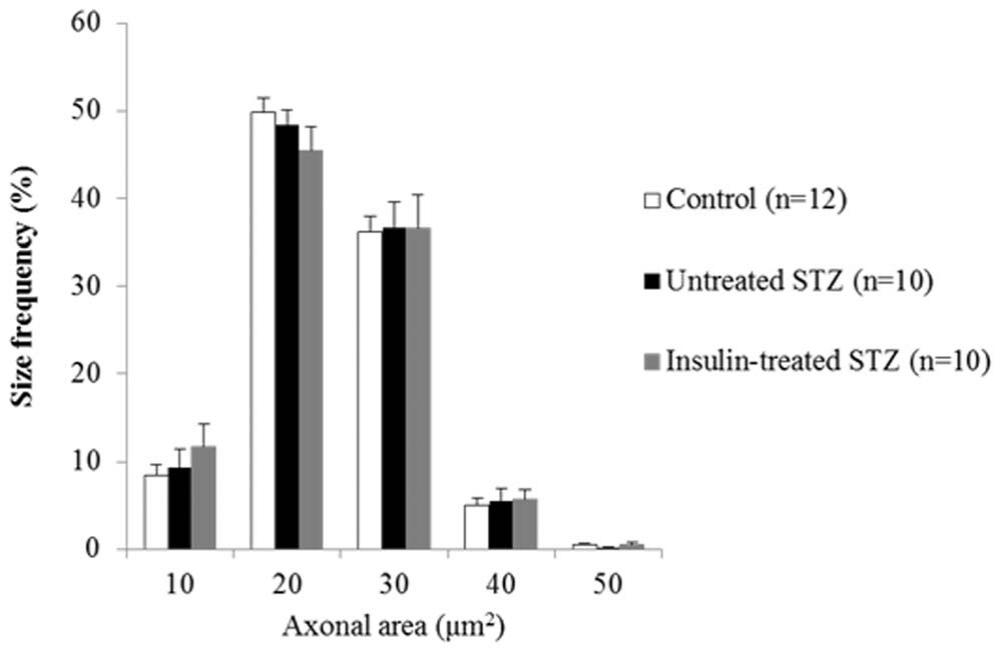
Size distribution histograms of intact myelinated axons in sural nerves from control (white bars), untreated STZ-induced diabetic (black bars), and insulin-treated STZ-induced diabetic (grey bars) rats. Data are means ± SE.

Fascicular area, myelinated fiber density, myelinated fiber area, axonal area, and the axon/myelin ratio of the sural nerves were not significantly different among the control, untreated and insulin-treated STZ-induced diabetic rats (Table 2). The size frequency distributions of myelinated axons did not differ among the three groups (Fig. 3).

**Table 2:** Morphometric findings of sural nerves from control, untreated and insulin-treated STZ-induced diabetic rats.

	Control	Untreated STZ-induced diabetic rats	Insulin-treated STZ-induced diabetic rats
*N*	12	10	10
Fascicular area (mm^2^)	0.071±0.004	0.070±0.003	0.076±0.002
MFD (10^3^/mm^2^)	15.0±0.5	15.3±0.6	14.6±0.6
Myelinated fiber area (µm^2^)	39.2±0.9	39.1±1.2	38.5±1.5
Axonal area (µm^2^)	19.0±0.4	18.9±0.6	18.8±0.9
Axon/myelin ratio	1.01±0.02	0.98±0.02	1.00±0.03

Data are means ± SE. Intact myelinated fibers were morphometrically analysed. MFD: myelinated fiber density.

### Footpad IENFD

**Figure 4:**
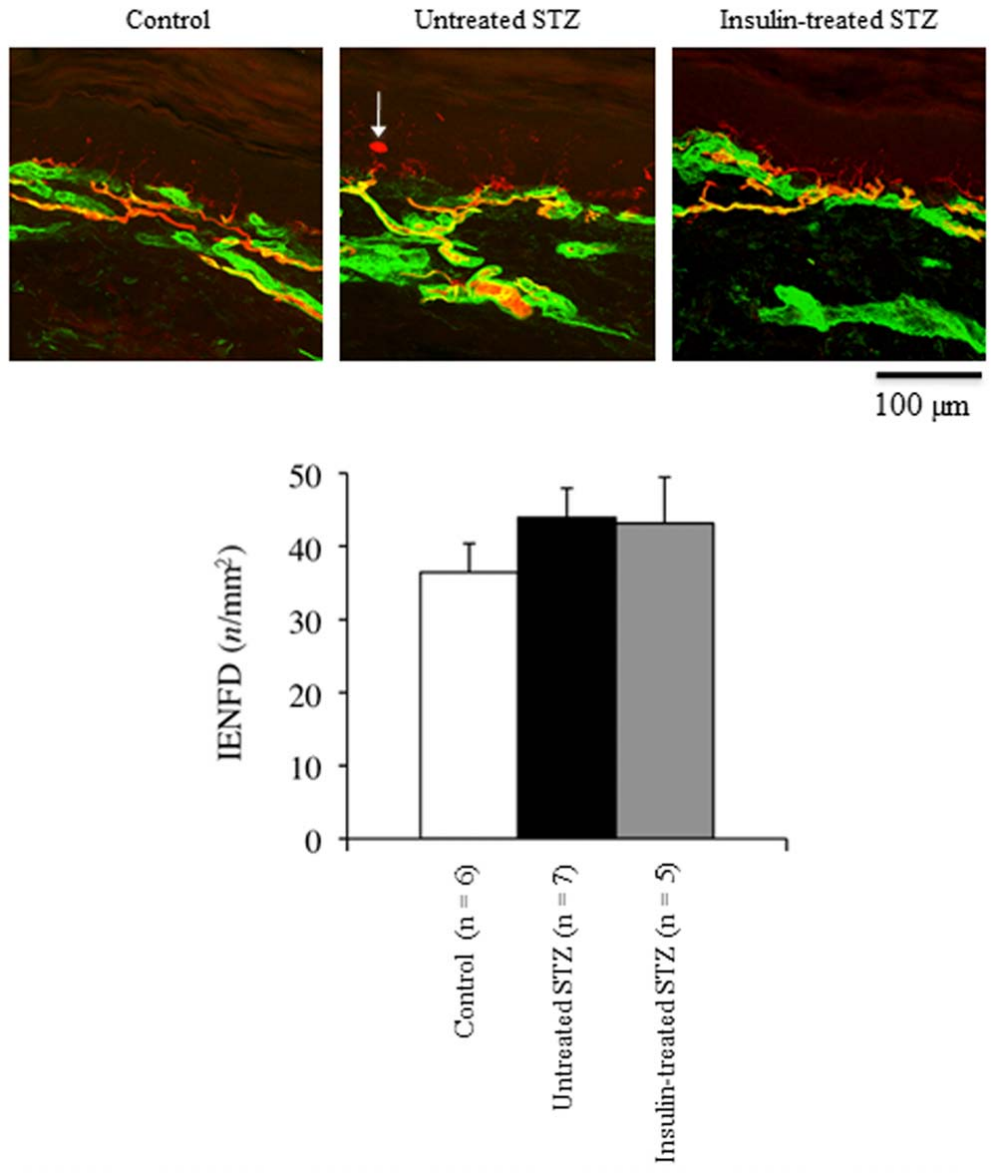
Representative confocal images of the epidermis and superficial dermis of footpads from control (white bars), untreated STZ-induced diabetic (black bars), and insulin-treated STZ-induced diabetic (grey bars) rats. Nerve fibers immunoreactive for PGP9.5 appear red or yellow. The epidermal basement membrane and subepidermal vasculature are immunoreactive for type IV collagen (green). The arrow indicates a PGP-immunoreactive Langerhans cell.

The IENFD tended to increase by 21% in the footpad of untreated STZ-induced diabetic rats compared with control rats; it was not affected by low-dose insulin administration (Fig. 4).

### Phosphorylated insulin receptor immunofluorescence in DRGs

**Figure 5:**
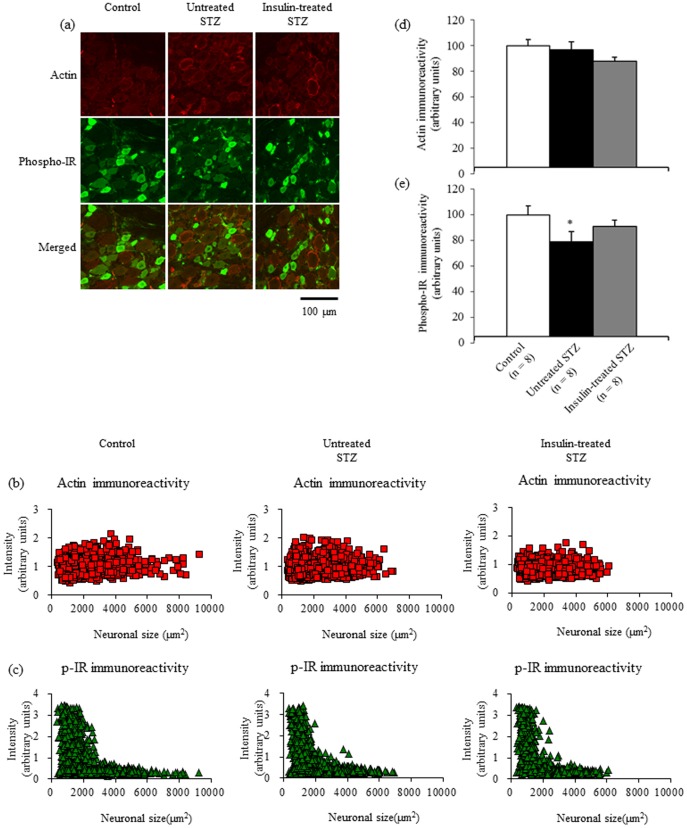
Double immunofluorescent labelling for actin and phosphorylated insulin receptor (a) from lumbar DRGs of control, untreated STZ-induced diabetic, and insulin-treated STZ-induced diabetic rats. Intensities of actin (b) and phosphorylated insulin receptor fluorescence (c) are displayed as a scatter plot against neuronal area. The mean pixel intensity of actin fluorescence in sensory neurons and satellite cells (d) does not differ among the three groups, whereas neuronal and satellite cell phosphorylated insulin receptor fluorescence intensity (e) is significantly decreased, by 21%, in untreated STZ-induced diabetic rats (black bars) and unchanged in insulin-treated STZ-induced diabetic rats (grey bars), compared with control rats (white bars). Data are means ± SE. Bar  =  100 µm. ^*^ *p*<0.05 vs. control rats. DRG: dorsal root ganglion; IR: insulin receptor.

Figure 5 shows the localization of immunoreactive actin and phosphorylated insulin receptors in DRGs. Intense actin immunoreactivity in DRGs was localized mainly to satellite cells, the walls of small vessels, and the perineurium, whereas intense phosphorylated insulin receptor immunoreactivity was localized mainly to DRG neurons smaller than 3000 µm^2^ in control rats (Fig. 5a, b, c). There appeared to be a decreased intensity of phosphorylated insulin receptor fluorescence in a subpopulation of neurons measuring between 1500 and 3000 µm^2^ in size in untreated STZ-induced diabetic rats, which was partially restored by insulin treatment (Fig. 5c). Neuronal and satellite cell nuclei were not immunoreactive for actin or phosphorylated insulin receptor. The mean pixel intensity of actin fluorescence in neurons and satellite cells (Fig. 5d) was comparable among the three groups, whereas that of phosphorylated insulin receptor fluorescence (Fig. 5e) was significantly decreased by 21% in untreated STZ-induced diabetic rats and unchanged in insulin-treated STZ-induced diabetic rats compared with control rats.

### Phosphorylated p44/42 MAPK immunofluorescence in DRGs

**Figure 6:**
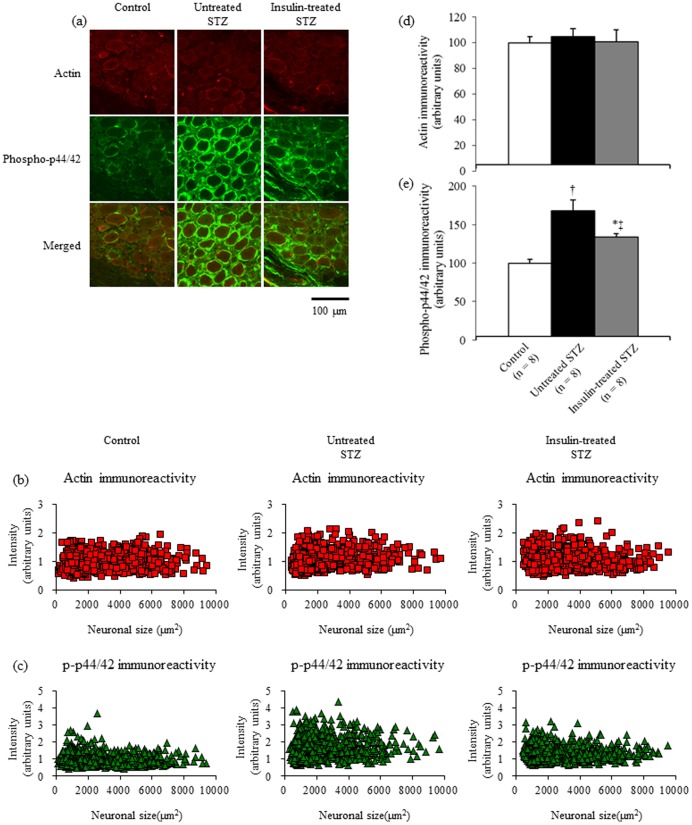
Double immunofluorescent labelling for actin and phosphorylated p44/42 (a) from lumbar DRGs of control, untreated STZ-induced diabetic, and insulin-treated STZ-induced diabetic rats. Intensities of actin (b) and phosphorylated p44/42 fluorescence (c) are displayed as a scatter plot against neuronal area. The mean pixel intensities of actin (d) in sensory neurons and satellite cells are unchanged among the three groups. Neuronal and satellite cell phosphorylated p44/42 (e) fluorescence intensity is significantly increased by 68% in untreated STZ-induced diabetic rats (black bars) compared with control rats (white bars), whereas it is significantly decreased by 50% in insulin-treated STZ-induced diabetic rats (grey bars) compared with untreated STZ-induced diabetic rats. Data are means ± SE. Bar  =  100 µm. ^*^ *p*<0.05 and ^†^ *p*<0.0001 vs. control rats; ^‡^ *p*<0.05 vs. untreated STZ-induced diabetic rats. DRG: dorsal root ganglion.

Figure 6 shows immunoreactive phosphorylated p44/42 MAPK localization in DRGs. Intense phosphorylated p44/42 MAPK immunoreactivity in DRGs was localized mainly to satellite cells, whereas phosphorylated p44/42 MAPK immunoreactivity was less intense in DRG neurons (Fig. 6a). Phosphorylated p44/42 MAPK immunoreactivity was not found in the neuronal or satellite cell nuclei. In scatter plot of actin (Fig. 6b) and phosphorylated p44/42 MAPK fluorescence intensity (Fig. 6c) versus neuronal area, phosphorylated p44/42 MAPK fluorescence intensity appeared to be increased in all DRG neurons from untreated STZ-induced diabetic rats compared with control and insulin-treated STZ-induced diabetic rats. The mean pixel intensities of actin fluorescence (Fig. 6d) in DRG neurons and satellite cells were unchanged among the three groups. Neuronal and satellite cell phosphorylated p44/42 fluorescence intensity was significantly increased by 68% in untreated STZ-induced diabetic rats compared with control rats, whereas it was significantly decreased by 50% in insulin-treated STZ-induced diabetic rats compared with untreated STZ-induced diabetic rats (Fig. 6e).

### Sciatic nerve insulin signaling

**Figure 7:**
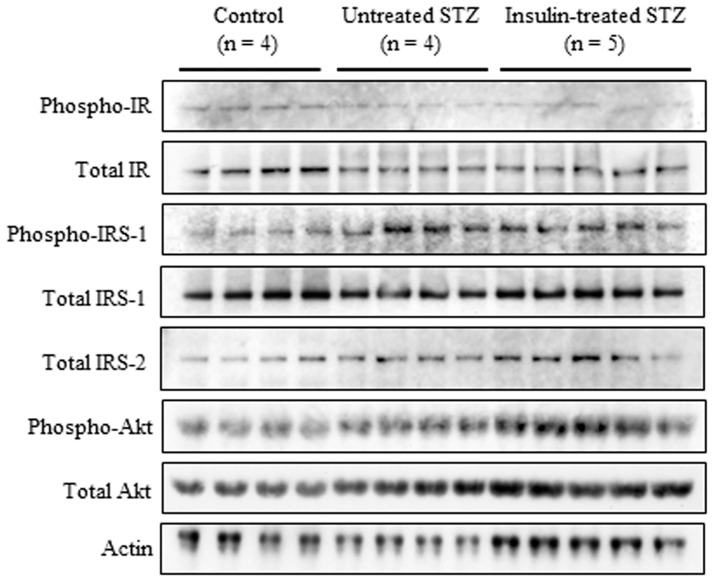
Western blot analyses of sciatic nerve IR, IRS-1, IRS-2, and Akt proteins, as well as of sciatic nerve IR, IRS-1, and Akt phosphorylation, in control (n = 4), untreated STZ-induced diabetic (n = 4), and insulin-treated STZ-induced diabetic (n = 5) rats. Proteins extracted from the sciatic nerve of one rat were immunoblotted with antibodies that recognized total IR, IRS-1, IRS-2, phosphorylated IR, phosphorylated IRS-1, phosphorylated Akt, or actin.

**Figure 8:**
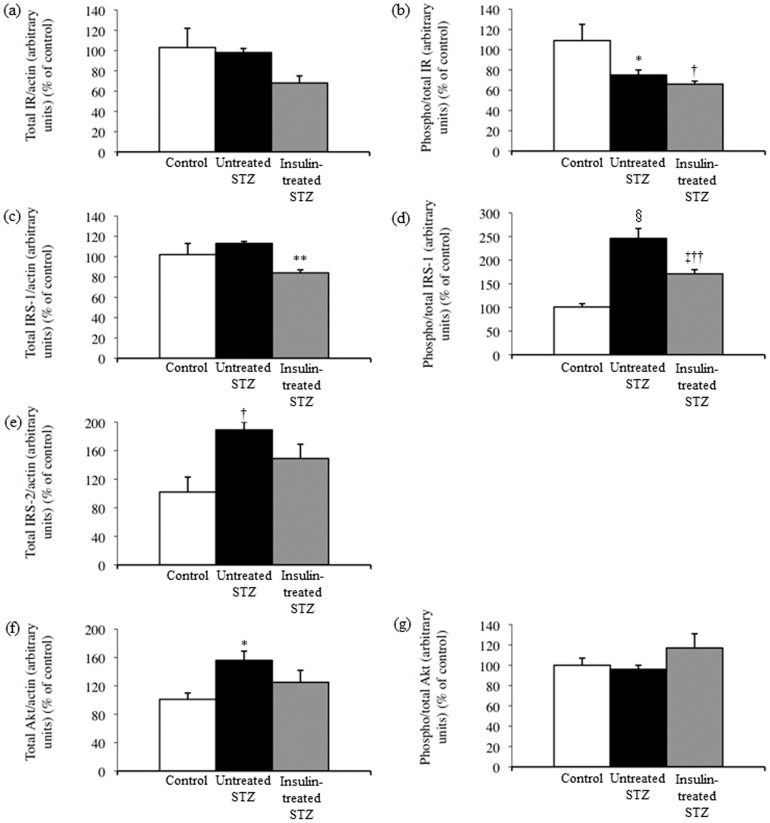
Western blot signals (Fig. 7) for total IR (a), IRS-1 (c), IRS-2 (e), and Akt (f) levels were normalized to actin levels, and those for phosphorylated IR (b), IRS-1 (d), and Akt (g) were normalized to their total protein levels. Values are means ± SE. White, black, and grey columns: control (n = 4), untreated STZ-induced diabetic (n = 4), and insulin-treated STZ-induced diabetic (n = 5) rats, respectively. ^*^ *p*<0.05, ^†^ *p*<0.01, ^‡^ *p*<0.005, and ^§^ *p*<0.0001 vs. control; ^**^ *p*<0.01, and ^††^ *p*<0.005 vs. untreated STZ-induced diabetic rats.

**Figure 9:**
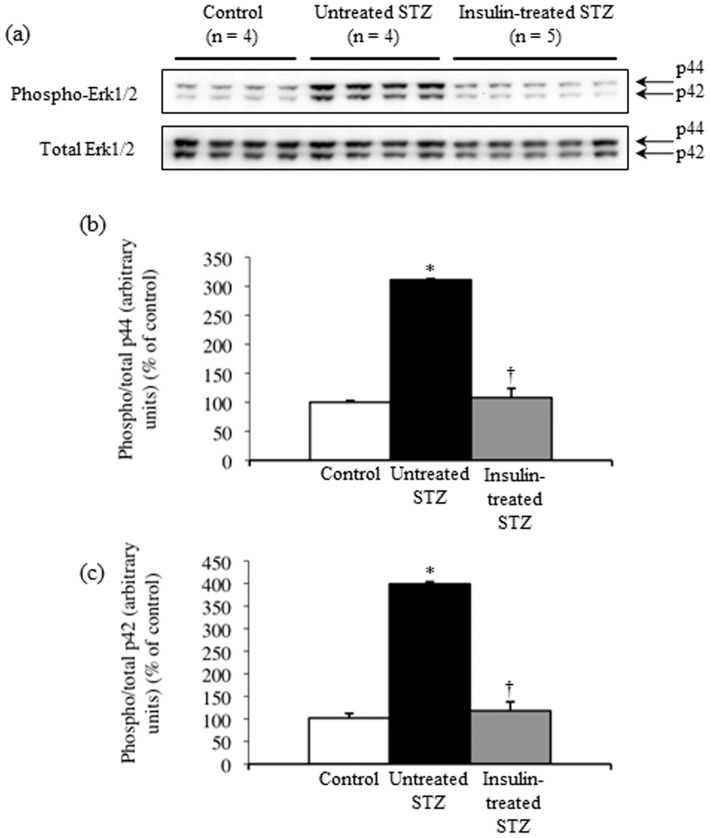
Representative blots show levels of sciatic nerve p44/42 MAPK total proteins and their phosphorylation in control (n = 4), untreated STZ-induced diabetic (n = 4), and insulin-treated STZ-induced diabetic (n = 5) rats (a). Proteins extracted from the sciatic nerve of one rat were immunoblotted with antibodies that recognized total p44/42 MAPK or phosphorylated p44/42 MAPK. Western blot signals for phosphorylated p44 (b) and p42 (c) were normalized to their total protein levels. Values are means ± SE. White, black, and grey columns: control (n = 4), untreated STZ-induced diabetic (n = 4), and insulin-treated STZ-induced diabetic (n = 5) rats, respectively. ^*^ *p*<0.0001 vs. control; ^†^ *p*<0.0001 vs. untreated STZ-induced diabetic rats.

The insulin receptor total protein level did not differ significantly among sciatic nerves from control, untreated and insulin-treated STZ-induced diabetic rats (Fig. 7, Fig. 8a). The level of phosphorylated insulin receptor relative to that of insulin receptor total protein was significantly decreased by 31% and 39%, respectively, in sciatic nerves from untreated and insulin-treated STZ-induced diabetic rats compared with control rats (Fig. 7, Fig. 8b). The IRS-1 total protein level was unchanged (Fig. 7, Fig. 8c), and the level of phosphorylated IRS-1 relative to that of IRS-1 total protein increased 2.5-fold in sciatic nerves from untreated STZ-induced diabetic rats compared with control rats (Fig. 7, Fig. 8d). The sciatic nerve IRS-1 total protein level and phosphorylation were decreased by 25% and 30%, respectively, in insulin-treated STZ-induced diabetic rats compared with untreated STZ-induced diabetic rats (Fig. 7, Fig. 8c, d). Sciatic nerve IRS-2 and Akt total protein levels increased by 84% and 54%, respectively, in untreated STZ-induced diabetic rats compared with control rats, whereas they were non-significantly decreased by 21% and 20%, respectively, in insulin-treated STZ-induced diabetic rats compared with untreated STZ-induced diabetic rats (Fig. 7, Fig. 8e, f). The level of phosphorylated Akt relative to that of Akt total protein remained unchanged among the three groups (Fig. 7, Fig. 8g). There were 3.1-fold and 4.0-fold increases in sciatic nerve p44 (Fig. 9a, b) and p42 (Fig. 9a, c) MAPK phosphorylation, respectively, in untreated STZ-induced diabetic rats compared with control rats. Low-dose insulin administration normalized the increased p44 and p42 MAPK phosphorylation.

## Discussion

The administration of low-dose insulin insufficient to affect overall glycemia to STZ-induced diabetic rats for up to 6 weeks improved the nociception dysfunction and sensory nerve conduction deficit without altering myelinated sensory fiber morphology and epidermal innervation. These functional benefits were associated with restoration of phosphorylated insulin receptor expression in sensory neurons and deactivation of p44/42 MAPK in sensory neurons and sciatic nerves. Downregulation of IRS-1 and -2 in sciatic nerves was also observed in insulin-treated STZ-induced diabetic rats. Therefore, it appears that an insulin deficiency rather than hyperglycemia induces aberrant neuronal insulin receptor signaling and contributes to sensory nerve dysfunction in type 1 diabetic neuropathy.

There are three main groups of MAPKs, the extracellular signal-regulated kinases (ERKs), the p38 kinases, and the c-jun N-terminal kinases (JNKs), and their pathways may be activated in injured nerves via distinct molecular and cellular mechanisms [18]. Of these, it is reported that sequential activation of ERK1 (p44 MAPK) and ERK2 (p42 MAPK) in the DRG neurons and satellite cells mediates pain after spinal nerve ligation in rats [19]. MAPKs might also be involved in responses to diabetes-derived cellular changes and be activated by hyperglycemia-induced oxidative stress in DRGs from STZ-induced diabetic rats [20]. Previous studies have reported increased basal ERK phosphorylation in the DRGs of STZ-induced diabetic rodents, but not in sciatic nerves [21], [22]. In addition, mechanical hyperalgesia has been shown to be correlated with an early increase in ERK, p38, and JNK phosphorylation in the spinal cord and dorsal root ganglion shortly after induction of diabetes by STZ in rats [23]. In the present study, insulin receptor phosphorylation decreased and p44/42 MAPK phosphorylation increased, both in the DRGs and sciatic nerves of STZ-induced diabetic rats. Furthermore, low-dose insulin decreased p44/42 MAPK phosphorylation to control levels along with the restoration of insulin receptor phosphorylation in the DRG and the downregulation of IRS-1, IRS-2, and Akt expressions in the sciatic nerve of STZ-induced diabetic rats. Therefore, the present findings are the first to indicate that an insulin deficiency per se induces impaired peripheral nerve insulin receptor signaling involving p44/42 MAPK activation associated with early nociceptive dysfunction in **STZ-induced diabetic rats**

Actin has often been used as a loading control in Western blot analysis. In the immunofluorescence analysis, we used actin as a control to check for equal background signals among different samples and found that actin immunoreactivity was localized mainly to satellite cells, the walls of small vessels, and the perineurium. This finding suggests that actin is not a suitable control for housekeeping gene expression in neurons. On the other hand, the scatter plot revealed a decrease in phosphorylated insulin receptor immunoreactivity in a specific subpopulation of small neurons with areas of ca. 1500–3000 µm^2^ in untreated STZ-induced diabetic rats, which was partially restored by insulin treatment (Fig. 5c). In addition, all DRG neurons from untreated STZ-induced diabetic rats showed an increase in phosphorylated p44/42 MAPK immunoreactivity, and insulin treatment ameliorated this abnormality (Fig. 6c). These novel findings suggest that insulin deficiency affects different subpopulations of sensory neurons via different molecular mechanisms with or without the suppression of insulin receptor phosphorylation and warrant further investigation to fully characterize biological effects of insulin in peripheral nerve.

In the present study, insulin treatment led to an insignificant increase in serum insulin levels from 0.17 ng/ml to 0.34 ng/ml and resulted in a significant decrease in serum free fatty acid levels and improvement in sensory nerve function, albeit with no significant reduction in hemoglobin A1c levels, in STZ-induced diabetic rats. This suggests that free fatty acid metabolism and sensory nerve function are more sensitive to the insulin therapy than glucose metabolism in this model. The mechanism that underlies such differential effects of insulin treatment remains unclear. One may claim that the observed increase in serum insulin levels is too small to produce any significant biological effects when the affinity of insulin receptors to insulin is taken into account [24], [25]. It is reported that the high-affinity insulin receptor (isoform A) binds insulin-like growth factor (IGF)-II with high affinity in fetal and cancer cells [25]-[27]. In addition, both the insulin receptor isoform A and isoform B are able to form hybrids with the IGF-I receptor in various rodent and human cell lines or hepatoblastoma cells: the hybrid receptor with the insulin receptor isoform A has an higher affinity for IGF-I and also binds IGF-II and insulin, whereas the hybrid receptor with the insulin receptor isoform B has a high affinity only for IGF-I. [24]. Thus, the regulation of insulin receptor isoform expression has important implications in the biological effects of insulin, IGF-I, and IGF-II. Although we have reported that the insulin receptor isoform A is preferentially expressed in sensory neurons [11], [12], no information is available on the expression of the hybrid receptors in sensory neurons.

Consistent with our previous findings [13], the present study demonstrated a decreased level of phosphorylated insulin receptor relative to that of insulin receptor total protein and unaltered insulin receptor total protein expression in the sciatic nerves of STZ-induced diabetic rats. The present study also found decreased neuronal expression of phosphorylated insulin receptor protein in this model. Since we recently reported that neuronal expressions of total and phosphorylated insulin receptor protein, as well as insulin receptor total protein expression in sciatic nerves, were all decreased in type 2 diabetic Zucker diabetic fatty (ZDF) rats [16], it is possible that the underlying mechanisms for impaired insulin receptor signaling in peripheral nerves differ between insulin-deficient type 1 and insulin-resistant type 2 diabetic animal models. This notion appears to be consistent with the previous report that demonstrated distinct functional, structural, and molecular abnormalities in peripheral nerves in the type 1 and type 2 diabetic rat models [4], [28].

STZ-induced diabetic animals have been used most extensively as an experimental model of diabetic neuropathy. In this model, hyperglycemia-induced metabolic alterations, such as increased polyol (sorbitol) pathway activity, reduced myo-inositol content, altered protein kinase C activity, and oxidative stress, have been claimed to be central to the pathogenesis of diabetic neuropathy. Based on this assumption, a number of clinical intervention trials have been performed. However, our understanding of human diabetic neuropathy remains incomplete. In this regard, peripheral nerve dysfunction in STZ-induced diabetic animals may correlate with insulin deficiency [5]-[7], [9], [10]. Nociceptive dysfunction in type 2 diabetic ZDF rats may also be independent of glycaemic status [29] and correlate with the presence or absence of hyperinsulinaemia that compensates for the insulin resistance [30]. More recent studies have reported that hyperinsulinaemia blunts insulin receptor signaling [31] and insulin-induced survival and outgrowth of neuritis in sensory neurons [32]. In conjunction with our previous study, this suggests that impaired peripheral nerve insulin receptor signaling in type 1 diabetic STZ-induced diabetic rats, which had different properties from those in type 2 diabetic ZDF rats, was altered by low-dose insulin. In particular, the present study is the first to present evidence of relationships among insulin deficiency, p44/42 MAPK activation, and peripheral nerve dysfunction in a rat model of type 1 diabetes. Since insulin has been shown to function both in the central and peripheral nervous systems [33], [34] and to improve peripheral nerve function in non-diabetic [35] and diabetic subjects [36], [37], independent of glycemic levels, insulin receptor signaling in peripheral nerves may deserve much greater attention when considering the unmet need to better understand and establish more effective therapeutic strategies for human diabetic neuropathy.

Quantification of intraepidermal nerve fibers has increasingly been used to evaluate the extent of involvement of unmyelinated sensory fibers [38]. In the present study, IENFD appeared to increase despite the early development of nociceptive dysfunction in STZ-induced diabetic rats, suggesting the development of a functional, but not structural, abnormality of small sensory fibers in this model. Although some researchers [39], [40] reported a reduction in IENFD in STZ-induced diabetic rats, the present finding is consistent with previous studies showing a trend toward increases in myelinated [41], [42] and unmyelinated fiber number/density [43], [44] in the peripheral nerves of STZ-induced diabetic rats. In humans, IENFD decreases in the early period of type 2 diabetes or even in prediabetes [45]-[48]. In addition, supervised exercise with or without diet counselling results in epidermal reinnervation and improves neuropathic symptoms in both diabetic [49] and prediabetic neuropathy [50]. Therefore, it is possible that physical inactivity negatively influences small sensory fiber function and structure. However, this hypothesis has yet to be tested in animal models.

In summary, insulin-deficient STZ-induced diabetic rats had distinct alterations in peripheral nerve insulin receptor signaling that differed from those in insulin-resistant ZDF rats. A low dose of insulin, insufficient to affect systemic glycemia, partially restored the impaired peripheral nerve insulin receptor signaling involving the deactivation of p44/42 MAPK and ameliorated peripheral sensory nerve dysfunction in STZ-induced diabetic rats. These findings support the notion that, besides hyperglycemia, insulin deficiency is involved in the pathogenesis of type 1 diabetic neuropathy.
